# So-Cheong-Ryong-Tang induces apoptosis through activation of the intrinsic and extrinsic apoptosis pathways, and inhibition of the PI3K/Akt signaling pathway in non-small-cell lung cancer A549 cells

**DOI:** 10.1186/s12906-015-0639-y

**Published:** 2015-04-10

**Authors:** Cheol Park, Su Hyun Hong, Gi-Young Kim, Yung Hyun Choi

**Affiliations:** Department of Molecular Biology, College of Natural Sciences, Dongeui University, 176 Eomgwangno Busanjin-gu, Busan, 614-714 Republic of Korea; Department of Biochemistry, Dongeui University College of Korean Medicine, 52-57, Yangjeong-ro, Busanjin, Busan, 614-052 Republic of Korea; Laboratory of Immunobiology, Department of Marine Life Sciences, Jeju National University, 102 Jejudaehak-ro, Jeju, 690-756 Republic of Korea; Anti-Aging Research Center and Blue-Bio Industry RIC, Dongeui University, 176 Eomgwangno Busanjin-gu, Busan, 614-714 Republic of Korea

**Keywords:** So-Cheong-Ryong-Tang, A549 cells, Apoptosis, Caspase, PI3K/Akt

## Abstract

**Background:**

So-Cheong-Ryong-Tang (SCRT), a traditional Korean medicine containing eight species of medicinal plant, has been used to treat patients with bronchial asthma and allergic rhinitis for hundreds of years; however, its anti-cancer potential is poorly understood. The present study was designed to evaluate the apoptotic effect of SCRT against human non-small-cell lung cancer (NSCLC) A549 cells.

**Methods:**

The effects of SCRT on cell growth and viability were evaluated by trypan blue dye exclusion and 3-(4, 5-dimethyl-thiazol-2-yl)-2, 5-diphenyl tetrazoliumbromide (MTT) assays, respectively. Apoptosis was detected using 4,6-diamidino-2-phenyllindile (DAPI) staining, agarose gel electrophoresis and flow cytometry. The protein levels were determined by Western blot analysis. Caspase activity was measured using a colorimetric assay.

**Results:**

SCRT treatment resulted in significantly decreased A549 cell growth and viability by induction of apoptosis. SCRT induced the translocation of pro-apoptotic Bax to the mitochondria, mitochondrial membrane permeabilization, cytochrome *c* release from mitochondria to cytosol, and activated caspase-9 and caspase-3. SCRT also increased death receptor-associated ligands and enhanced the activation of caspase-8 and cleavage of its substrate Bid. However, the pan-caspases inhibitor significantly blocked the SCRT-induced apoptosis, suggesting that it is a caspase-dependent pathway. In addition, SCRT suppressed the phosphorylation of phosphoinositide 3-kinase (PI3K) and Akt, and treatment with a potent inhibitor of PI3K further increased the apoptotic activity of SCRT.

**Conclusions:**

These findings suggest that SCRT may play its anti-cancer actions partly through a suppression of the PI3K/Akt signal pathway in A549 cells, and further *in vivo* studies on the potential of SCRT for prevention and therapy of NSCLCs are warranted.

## Background

Non-small-cell lung cancer (NSCLC), accounting for about 80% of human lung cancers, is one of the leading causes of cancer deaths worldwide. Therapeutic strategies for lung cancer treatment include surgery, radiotherapy, chemotherapy, and targeted and combined therapies. Despite advances in treatment, the poor prognosis is largely attributable to the inherent or acquired resistance in cancer cells against conventional chemotherapy [1,2]. Although, many studies have reported potential chemotherapeutic effects of novel compounds, novel agents with proven efficacy and minimal toxicity are urgently required for the treatment of NSCLC.

The progression of cancer is characteristically linked to a resistance to apoptosis, a major type of cell death that occurs when DNA damage is irreversible. Apoptosis is characterized by distinct morphological and biochemical changes such as cell shrinkage, membrane blebbing, chromatin condensation, activation of proteases, and externalization of phosphatidylserine [3,4]. Therefore, agents targeting the apoptosis pathway without affecting normal cells play crucial roles as potential drug targets in cancer treatment. In mammalian cells, apoptosis may be initiated in two ways, via an intrinsic (mitochondrial-mediated) or extrinsic (death receptor (DR)-mediated) pathway that involves the activation of caspases [5,6]. The former is activated by the release of pro-apoptotic factors such as cytochrome *c* from the mitochondrial matrix following the loss of inner mitochondrial membrane integrity and activation of caspase-9. The latter is initiated by the binding of extracellular death ligands to their cell-surface death receptors leading to the activation of caspase-8. The extrinsic pathway can crosstalk to the intrinsic pathway through the caspase-8-mediated cleavage of Bid, a member of the Bcl-2 family of proteins, which ultimately leads to apoptosis [7,8].

For thousands of years, herbal medicines have been used seemingly safely and effectively to treat and alleviate various diseases in Asia, including Korea, China, and Japan. Recently, there has been increasing interest in the pharmacological activity of traditional medicines, and numerous studies support their potential clinical benefit for diseases that are difficult to treat [9,10]. Typical traditional Korean medicines derived from ancient Chinese herbal medicines and consist of at least four 4 components that are mixed to minimize side effects, maximize medical effects, and improve the patient’s quality of life. So-Cheong-Ryong-Tang (SCRT), also known as Xiao-Qing-Long-Tang in China and Sho-Seiru-To in Japan, is an aqueous polyherbal formulation, consisting of eight species of medicinal herb. SCRT has been used to treat bronchial asthma and allergic rhinitis for hundreds of years in Asian countries [11]. Studies regarding its pharmacological action have shown an inhibition of histamine release and degranulation of mast cells [12], reduced serum IgE level in allergic rhinitis patients [13,14], decreased allergen-induced bronchial inflammation [15], the growth and differentiation of basophils [16] and passive cutaneous anaphylaxis [17]. Recently, Byun et al. [18] demonstrated that SCRT improved the quality of life and reduced the symptoms of illness in common cold patients, and it was also found that SCRT exhibits immunomodulative and anti-inflammatory activities [19-21]. However, despite its valuable effects, little is known about the anti-cancer potential of SCRT. In this study, as a part of our search for novel biologically active substances for the prevention and treatment of cancer from traditional Korean medicine we evaluated whether SCRT could inhibit cell growth and trigger apoptosis in NSCLC A549 cells.

## Methods

### Reagents and antibodies

Dulbecco’s modified eagle’s medium (DMEM), fetal bovine serum (FBS), penicillin, streptomycin, and trypsine-ethylene diamine tetraacetic acid (EDTA) were purchased from Gibco-BRL (Gaithersburg, MD, USA). 3-(4,5-dimethyl-2-thiazolyl)-2,5-diphenyl-2H-tetrazolium bromide (MTT), 4,6-diamidino-2-phenyllindile (DAPI), phenol:chloroform:isoamylalcohol, ethidium bromide (EtBr), RNase A, dithiothreitol (DTT), bovine serum albumin (BSA), propidium iodide (PI), paraformaldehyde and 5,5′,6,6′-tetrachloro-1,1′,3,3′-tetraethyl-imidacarbocyanine iodide (JC-1) were purchased from Sigma-Aldrich Chemicals (St. Louis, MO, USA). *N*-benzyloxycarbonyl-Val-Ala-Asp-fluoromethylketone (z-VAD-fmk), a pan-caspase inhibitor, and LY294002, a selective inhibitor of phosphatidylinositol 3-kinase (PI3K), were obtained from Calbiochem (San Diego, CA, USA) and Cell Signaling Technology, Inc. (Danvers, MA, USA), respectively. Annexin V-fluorescein isothiocyanate (FITC) and caspase activity assay kits were purchased from R&D Systems (Minneapolis, MN, USA). DNA ladder size marker and enhanced chemiluminescence (ECL) kit were purchased from Invitrogen (Carlsbad, CA, USA) and Amersham Corp. (Arlington Heights, IL, USA), respectively. A mitochondrial fractionation kit was obtained from Active Motif (Carlsbad, CA, USA). Primary antibodies were purchased from Santa Cruz Biotechnology (Santa Cruz, CA, USA), Chemicon (Temecula, CA, USA) and Sigma-Aldrich. Peroxidase-labelled donkey anti-rabbit and sheep anti-mouse immunoglobulin were purchased from Amersham. All other chemicals not specifically mentioned here were purchased from Sigma-Aldrich.

### Preparation of SCRT extract

SCRT, containing eight species of medicinal plant (Table 1), was obtained from Dongeui Oriental Hospital at the Dongeui University College of Korean Medicine (Busan, Republic of Korea). Those eight herbs were authenticated by Professor Su Hyun Hong, Department of Biochemistry, Dongeui University College of Korean Medicine. Each herb in SCRT was cut into small pieces, ground to a fine powder, passed through a 20-mesh sieve, and then mixed with the others to yield at total sample of 40 g in the ratios in shown Table 1. The mixture was extracted with 500 ml of boiling water for six hours. The extracted liquid was filtered twice to remove insoluble materials. The filtered liquid was lyophilized and then crushed into a thin powder. The yield of dried extract from starting crude material was 9.72 g (24.3%, w/w). The extracts were resuspended in distilled water to a final concentration of 100 mg/ml (extract stock solution), and then diluted with media to the desired concentration prior to use. The voucher specimens (accession number DEU-30) have been deposited at a publicly available Natural Resource Bank of Dongeui University College of Oriental Medicine.

**Table 1:** **Components of So-Cheong-Ryong-Tang (SCRT) extract granules**

**Herbal medicine (pharmacognostic nomenclature)**	**Raw material amount (g/%)**
*Ephedra sinica* Stadf. (Ephedrae Herba)	6.0 (14.3)
*Paeonia lactiflora* Pall. (Paeoniae Radix)	6.0 (14.3)
*Schizandra chinensis* (Turcz.) Ball. (Schizandrae Fructus)	6.0 (14.3)
*Pinellia ternata* (Tenore et Breit. (Pinelliae Rhizoma)	6.0 (14.3)
*Asiarum sieboldii* F. Maekawa (Asiasari *Radix*)	4.0 (9.5)
*Zingiber officinale* Rosc. (Zingiberis Rhizoma)	4.0 (9.5)
*Cinnamomum cassia* Blume. (Cinnamomi Ramulus)	4.0 (9.5)
*Glycyrrhiza glabra* L. (Glycyrrhizae Radix)	4.0 (9.5)
Total amounts	40 (100)

### Cell culture

The human NSCLC A549 cells and WI-38 human fetal lung fibroblasts were purchased from the American Type Culture Collection (Manassas, VA, USA) and maintained in DMEM supplemented with 10% FBS, 1% L-glutamine and penicillin/streptomycin. The cells were cultured in an incubator with 5% CO_2_ at 37°C.

### Cell growth and cell viability assay

Cell growth was assessed using the trypan blue dye exclusion assay. In brief, cells (2 × 10^4^ cells/well) were seeded in 6-well plates. After treatment with the indicated concentrations of the SCRT for the indicated times, the cells were trypsinized and viable cells were counted by trypan blue dye exclusion using a hemocytometer under an inverted microscope (Carl Zeiss, Jena, Germany). Cell viability was determined using the MTT assay. In brief, cells (2 × 10^4^ cells/well) were seeded in 24-well plates and exposed to the extracts of SCRT for the indicated times. After treatment, 5 mg/ml MTT solution was added, followed by 3 h incubation at 37°C in the dark, and the media was then removed. The formazan precipitate was dissolved in dimethyl sulfoxide, and absorbance of the formazan product was measured at a wavelength of 540 nm with an enzyme-linked immunosorbent assay (ELISA) reader (Molecular Devices, Sunnyvale, CA, USA). For the morphological study, cells were photographed directly using an inverted microscope (Carl Zeiss, Oberkochen, Germany).

### Nuclear staining with DAPI

For the assessment of apoptosis, morphological changes of nuclei were visualized following DNA staining by the fluorescent dye, DAPI. The cells were fixed with 3.7% paraformaldehyde for 20 min at room temperature and washed with phosphate-buffered saline (PBS). Cells were then stained with 2.5 μg/ml DAPI solution for 10 min at room temperature. The cells were washed twice with PBS and stained nuclei were observed using a fluorescence microscope (Carl Zeiss).

### DNA fragmentation assay

After treatment with SCRT, cells were lysed in a buffer containing 10 mM Tris (pH 7.4), 150 mM NaCl, 5 mM EDTA and 0.5% Triton X-100 for 30 min on ice. Lysates were vortexed and cleared by 19,000 g centrifugation for 30 min at 4°C. Fragmented DNA in the supernatant was extracted with an equal volume of neutral phenol:chloroform:isoamylalcohol (25:24:1, v/v/v), followed by electrophoretic analysis on 1.5% agarose gels containing 0.1 μg/ml EtBr.

### Flow cytometric detection of apoptosis

To analyze cell cycle profiles, cells were harvested, washed twice with ice-cold PBS, fixed with 75% ethanol at 4°C for 30 min, and washed in PBS with 0.1% BSA. The cells were then incubated with 1 U/ml of RNase A (DNase free) and 10 μg/ml of PI overnight at room temperature in the dark. Cells were analyzed using a FACSCalibur flow cytometer (Becton Dickenson; San Jose, CA). The level of apoptotic cells containing sub-G1 DNA content was determined as a percentage of the total number of cells. The cells were also stained with 5 μl of annexin V-FITC and 5 μl of PI in each sample. After at 15 min incubation at room temperature in the dark, the degree of apoptosis was quantified as a percentage of the Annexin V-positive and PI-negative (annexin V^+^/PI^−^ cells) cells by a flow cytometer [22].

### Western blot analysis

Cells were harvested and lysed with lysis buffer (20 mM sucrose, 1 mM EDTA, 20 μM Tris-Cl, pH 7.2, 1 mM DTT, 10 mM KCl, 1.5 mM MgCl_2,_ and 5 μg/ml aprotinin) for 30 min at 4°C. The mixtures were centrifuged at 12,000 g for 10 min at 4°C, and the supernatants were collected as whole-cell extracts. In a parallel experiment, the mitochondrial and cytosolic fractions were isolated using a mitochondrial fractionation kit according to the manufacturer’s instructions. The protein content was determined using the Bio-Rad protein assay reagent (Bio-Rad, Hercules, CA, USA) and BSA as a standard according to the manufacturer’s instructions. Equal amounts of protein were separated electrophoretically using sodium dodecyl sulfate (SDS)-polyacrylamide gels transferred to nitrocellulose membranes (Amersham). The membranes were soaked in blocking buffer (5% skimmed milk) and incubated overnight with primary antibodies followed by horseradish peroxidase conjugated antibodies at room temperature. Detection of specific proteins was carried out with an ECL Western blotting kit according to the manufacturer’s instructions.

### Determination of caspase activity

The activity of caspase-like protease was measured using a caspase activation kit according to manufacturer’s protocol. The kits utilize synthetic tetrapeptides labeled with p-nitroanilide (pNA) linked to the end of the caspase-specific substrate. To evaluate caspase activity, cell lysates were prepared after treatment with SCRT and incubated with the supplied reaction buffer and the colorimetric substrates at 37°C for 2 h in the dark. The cleavage of the peptide by the caspase releases the chromophore pNA, which can be quantified spectrophotometrically at a wavelength of 405 nm using an ELISA reader.

### Measurement of mitochondrial membrane potential (MMP, Δψm)

The values of MMP were determined using the dual-emission potential-sensitive probe, JC-1, which is internalized and concentrated by respiring mitochondria and can therefore reflect changes in MMP in live cells. Cells were collected and incubated with 10 μM of JC-1 for 30 min at 37°C in the dark. After the JC-1 was removed, the cells were washed with PBS to remove any unbound dye and the amount of JC-1 retained by 10,000 cells per sample was measured at 488 nm and 575 nm using a flow cytometer.

### Statistical analysis

The data are reported as the mean ± standard deviation (SD) of three independent experiments. A one-way ANOVA was performed to determine statistical significance. Significant differences were established at *p* <0.05.

## Results

### Effects of SCRT on A549 cell growth and viability

**Figure 1:**
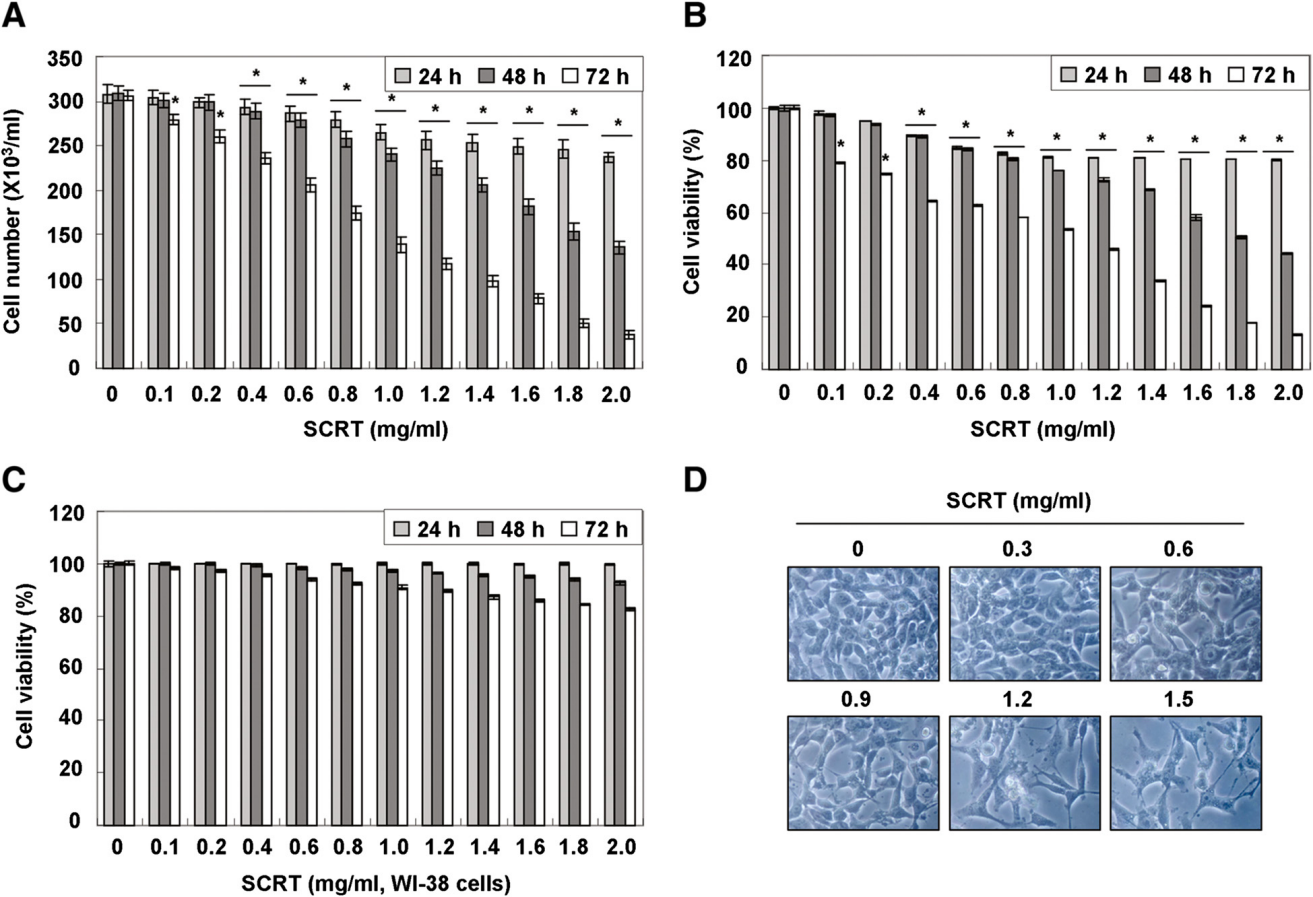
**SCRT inhibits cell viability and induces morphological changes in A549 cells. (A, B and C)** A549 and WI-38 cells were treated with various concentrations of SCRT for the indicated times. The cell viability was measured by the trypan blue exclusion method **(A)** and the metabolic-dye-based MTT assay **(B and C)**. Each point represents the mean ± SD of three independent experiments. The significance was determined by the Student’s *t*-test (*p < 0.05 vs. untreated control). **(D)** Cells were treated with the indicated concentrations of SCRT for 72 h and photographed under an inverted microscope (original magnification 200x).

**Figure 2:**
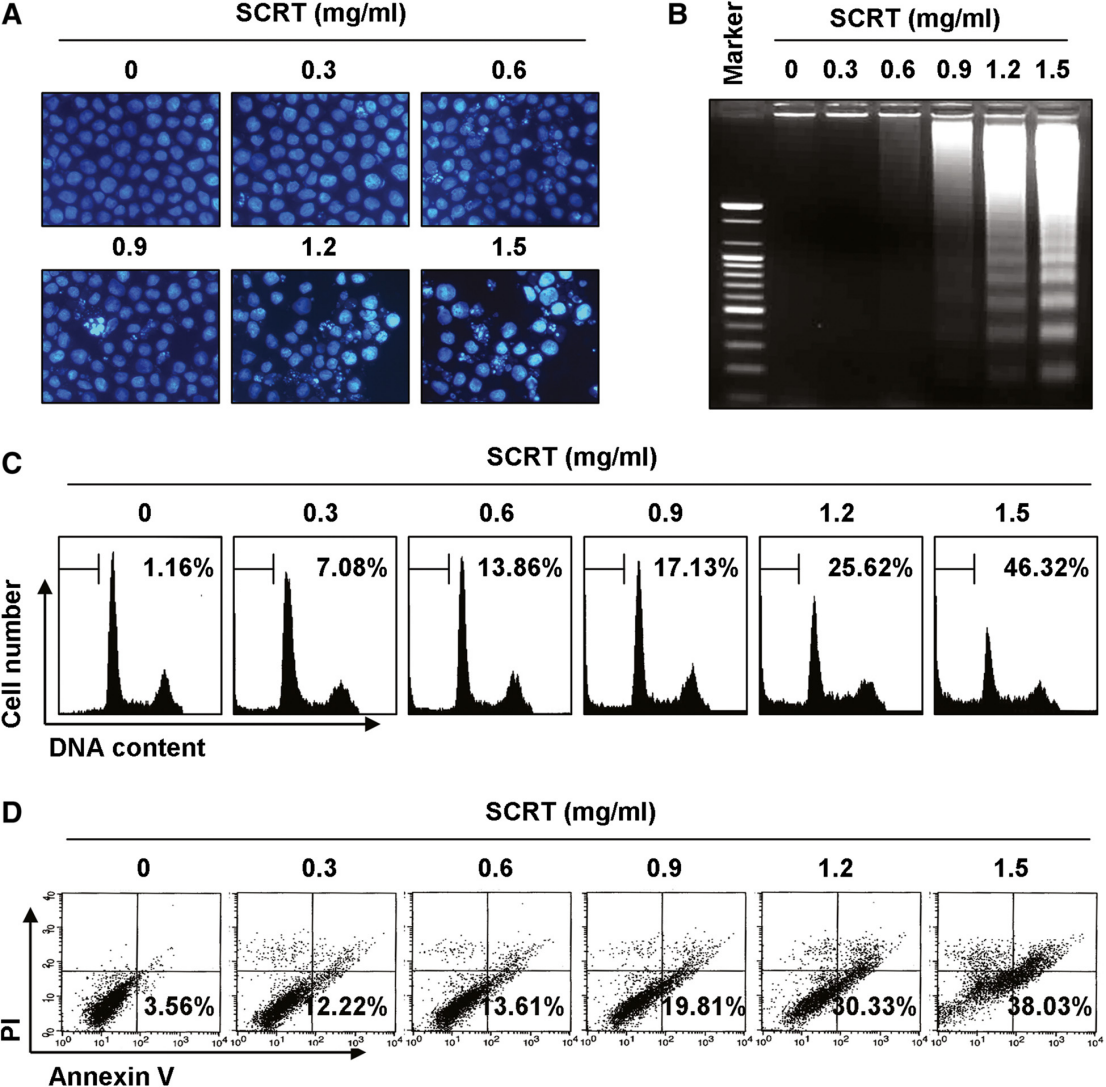
**SCRT induces apoptosis in A549 cells. (A)** Cells were treated with the indicated concentrations of SCRT for 72 h, fixed, and stained with DAPI solution. The stained nuclei were observed under a fluorescent microscope (original magnification 400x). **(B)** For the analysis of DNA fragmentation, genomic DNA from cells grown under the same conditions as **(A)** was extracted, separated by 1.5% agarose gel electrophoresis, and visualized under UV light after staining with EtBr. The DNA marker indicates the size of the fragments of the DNA ladder. **(C)** To quantify the degree of apoptosis induced by SCRT, cells were evaluated by a flow cytometer for sub-G1 DNA content, which represents the cells undergoing apoptotic DNA degradation. **(D)** The cells were stained with annexin V-FITC and PI, and the percentages of apoptotic cells (annexin V^+^ cells) were then analyzed using flow cytometric analysis. **(C and D)** The data is the mean of the two different experiments.

To examine the effect of SCRT on the cell growth and viability of A549 cells, cells were treated with various concentrations of SCRT for the indicated times and the assay, and then the trypan blue dye exclusion and MTT assays were performed. Our data indicated that SCRT exhibited cytotoxic effects in a dose- and time-dependent manner (Figure 1A and B), which was associated with significant morphological changes, including extensive cytosolic vacuolization and the appearance of irregular cell membrane buds (Figure 1D). However, the presence of SCRT did not inhibit the cell viability of WI-38 cells significantly (Figure 1C), indicating that SCRT has not cytotoxic effect to the normal lung fibroblast. We next examined the DAPI staining to determine whether the cytotoxic effect of SCRT was mediated by apoptosis. As shown in Figure 2A, when the SCRT concentration increased, more and more cells exhibited the morphological characteristics of apoptosis, such as chromatin condensation and apoptotic body formation. In addition to cell morphology, the fragmentation of chromatin into units of single or multiple nucleosomes was also examined with using agarose gel electrophoresis to observe the DNA ladder formation. As shown in Figure 2B, there was no evidence of oligonucleosomal DNA ladder in the control group; however, the DNA ladder became more evident in the SCRT groups in a dose-dependent fashion, indicating an increasing amount of apoptotic cells.

To measure apoptotic cell death upon SCRT treatment, we stained cells with PI and cycle profiles were analyzed using a flow cytometer. The results after PI staining showed that SCRT increased the percentage of cells in the apoptotic hypodiploid sub-G1 phase in a dose-dependent manner (Figure 2C). Annexin V-FITC/PI double staining was also performed to assess the rate of apoptotic cell death, and a significant degree of apoptosis was detected in the SCRT-treated A549 cells, as shown by an increased phosphatidyl exposure using annexin V-FITC (Figure 2D). The results we obtained corresponded very well with our cell morphology observations and DNA fragmentation analysis, indicating the cytotoxic effects observed in response to SCRT are associated with the induction of apoptosis in A549 cells.

### Activation of caspases by SCRT in A549 cells

**Figure 3:**
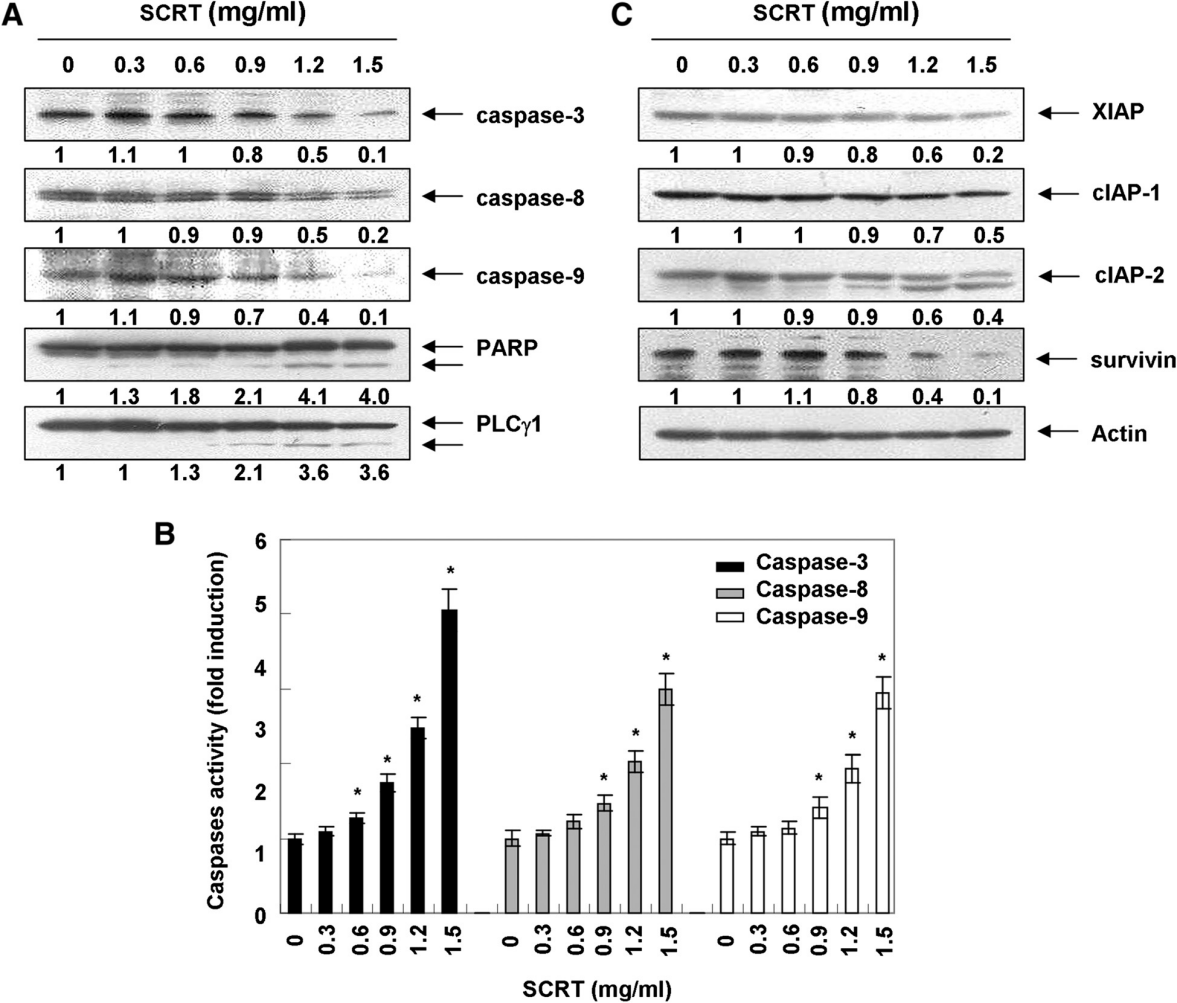
**Activation of caspases, degradation of PARP and PLC-γ1, and down-regulation of the IAP family proteins by SCRT in A549 cells. (A and C)** Cells were treated with the indicated concentrations of SCRT for 72 h. The cells were lysed, and the cellular proteins were visualized using the indicated antibodies and an ECL detection system. Actin was used as an internal control. The experiment was repeated three times and similar results were obtained. The relative ratios of expression in the results of the Western blotting were presented at the bottom of each of the results as relative values of the actin expression. **(B)** After 72 h incubation with the indicated concentrations of SCRT, the cells were lysed, and aliquots (50 μg protein) were assayed for *in vitro* caspase-3, −8 and −9 activity using DEVD-pNA, IETD-pNA and LEHD-pNA as substrates, respectively, at 37°C for 1 h. The released fluorescent products were measured. The data are expressed as the mean ± SD of three independent experiments. The significance was determined by the Student’s *t*-test (*p < 0.05 vs. untreated control).

The activation of caspases plays a very important role in apoptosis, and the activation of individual caspases is can either be an autocatalytic process or triggered by other members of the caspase family in a cascade fashion. Among several caspases, caspase-3 is downstream of both the extrinsic and intrinsic pathways, which include caspase-8 and −9 [4,5,8]. Therefore, we investigated whether SCRT activated any of these pathways using Western blot analysis and a colorimetric caspase activity assay. Although the active forms of caspases-8 and −9 were not observed, levels of the pro-forms of caspase-8, and −9, initiator caspases of extrinsic and intrinsic apoptosis pathways, respectively, were down-regulated while their *in vitro* activities were significantly increased in SCRT-treated A549 cells in a dose-dependent manner. These activities were accompanied by the activation of caspase-3 and the cleavage of poly(ADP-ribose) polymerase (PARP) and phospholipase C (PLC)-γ1, intracellular substrates for activated caspase-3 [23,24] (Figure 3A and B). These results indicated that SCRT activates initiator and executioner caspases involved in both the extrinsic and the intrinsic pathways. In addition, SCRT treatment down-regulated or cleaved the proteins of inhibitor of the apoptosis proteins (IAP) family, such as anti-X-linked inhibitor of apoptosis protein (XIAP), cellular inhibitor of apoptosis (cIAP)-1, cIAP-2 and survivin (Figure 3C), which bind to caspases and lead to their inactivation [25,26], indicating that SCRT-induced activation of caspases was associated with the reduction of IAP family proteins in A549 cells.

**Figure 4:**
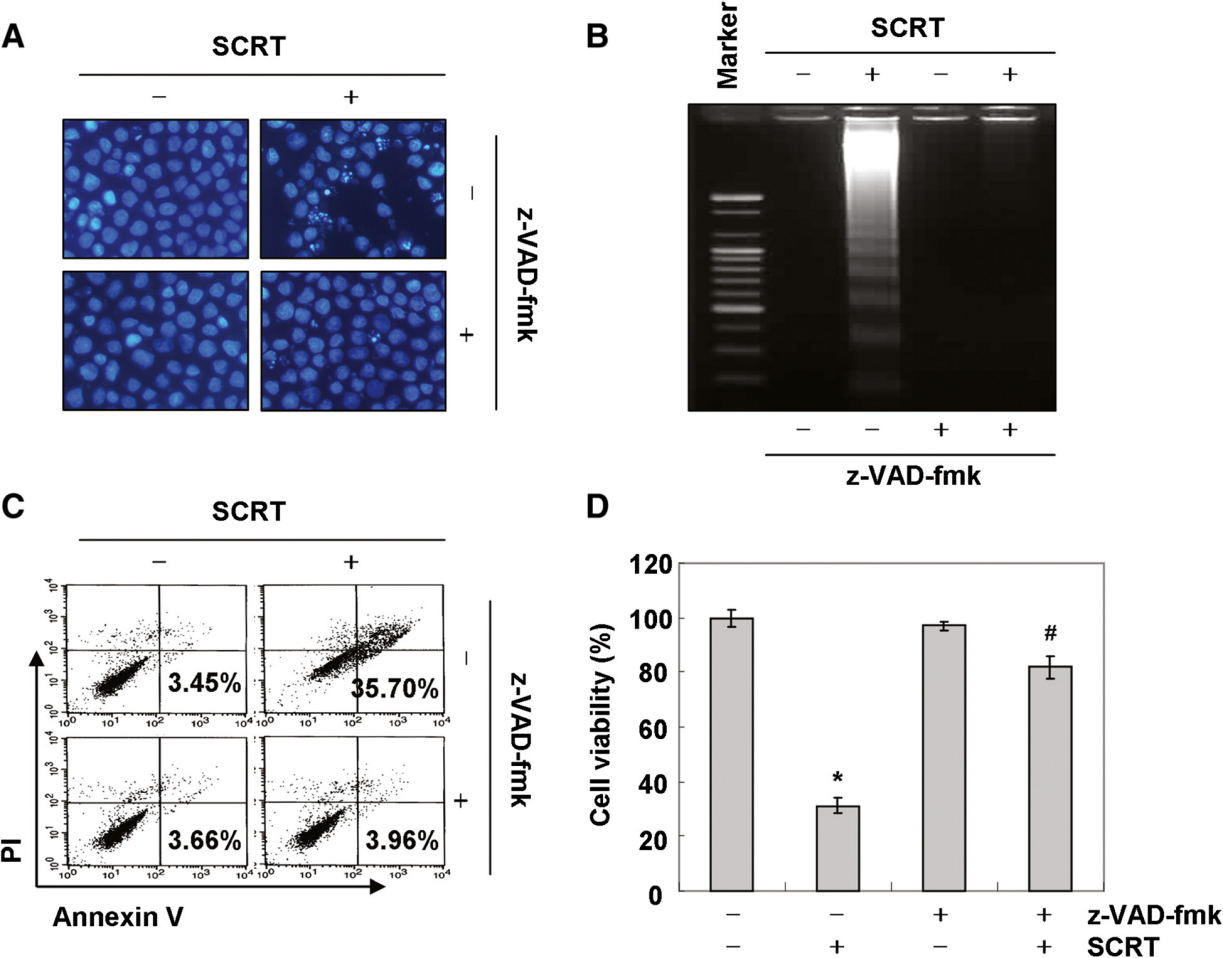
**Pan-caspase inhibitor z-VAD-fmk alleviates SCRT-induced apoptosis in A549 cells. (A)** Cells were incubated with 1.5 mg/ml of SCRT for 72 h after 1 h pretreatment with z-VAD-fmk (50 μM). After staining with DAPI solution, the nuclei were observed under a fluorescent microscope (original magnification 400x). **(B)** The genomic DNA from cells was extracted, separated by agarose gel electrophoresis, and visualized under UV light after staining with EtBr. **(C)** The percentages of apoptotic cells (annexin V^+^ cells) were analyzed using flow cytometric analysis. The data is the mean of the two different experiments. **(D)** Cell viability was determined by MTT assays. Each point represents the mean ± SD of three independent experiments. The significance was determined using Student’s *t*-test (*p < 0.05 vs. untreated control; ^#^ *p* < 0.05 vs. SCRT-treated cells).

To confirm caspase activation’s role in SCRT-induced apoptosis, cells were pre-incubated with a pan-caspase inhibitor, z-VAD-fmk, for 1 h and then treated with SCRT. As shown in Figure 4A and B, pre-treatment of A549 cells with z-VAD-fmk significantly inhibited apoptotic body formation, nuclear shrinkage, and DNA fragmentation following treatment with SCRT. Furthermore, z-VAD-fmk also decreased the accumulation of annexin V-FITC stained cells and increased cell viability in SCRT-treated A549 cells (Figure 4C and D), indicating that SCRT induces caspase-dependent apoptosis in A549 cells.

### Mitochondrial translocation of Bax and loss of MMP by SCRT in A549 cells

**Figure 5:**
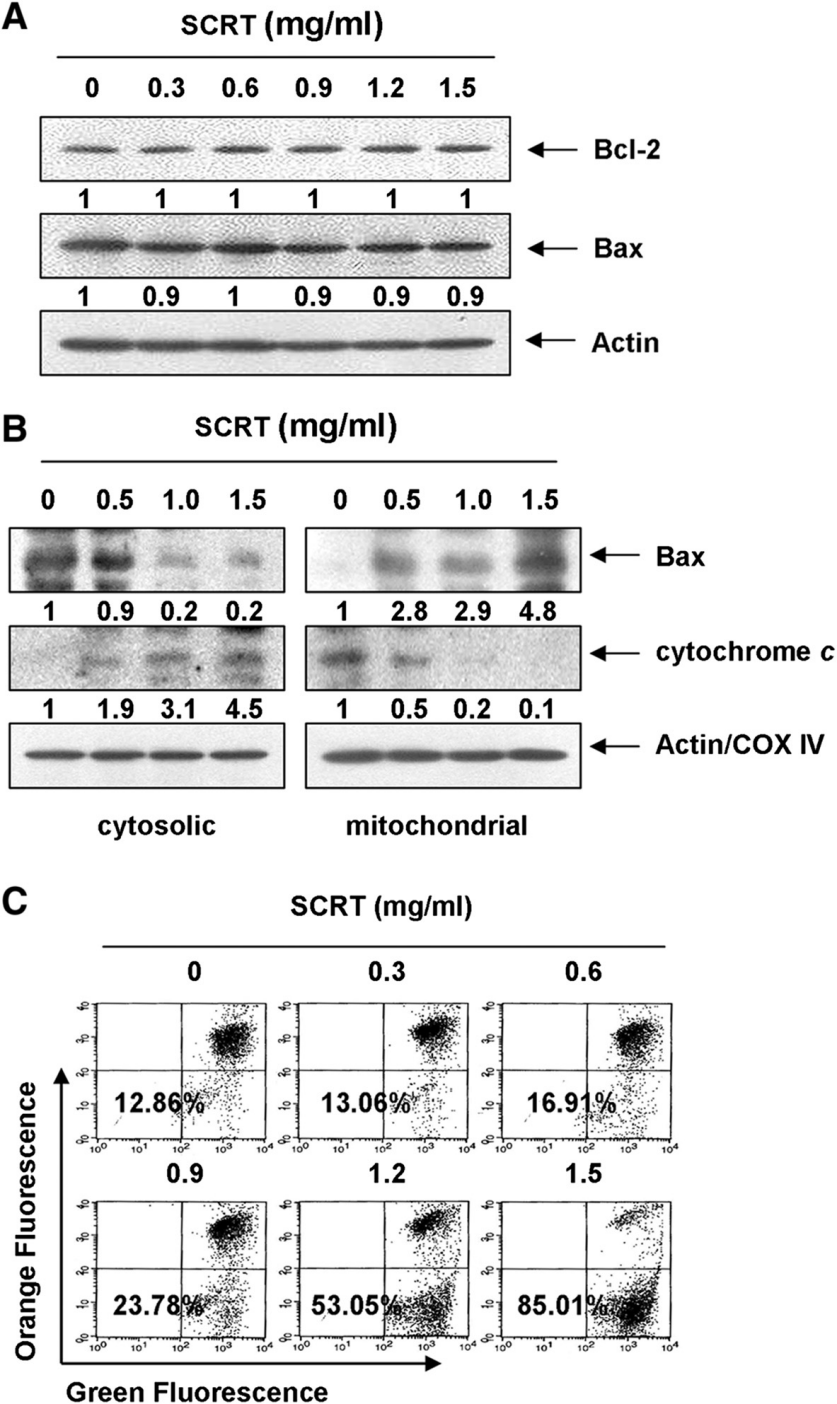
**Effects of SCRT on the expression of Bcl-2, Bax and cytochrome** ***c*** **, and values of MMP in A549 cells. (A)** Cells were treated with SCRT for 72 h, and aliquots containing total proteins were subjected to SDS-polyacrylamide gels followed by immunoblot analysis with specific antibodies. **(B)** The cytosolic and mitochondrial proteins were extracted from cells and analyzed by Western blotting using the indicated antibodies. Actin and cytochrome oxidase IV (COX IV) were used as internal controls for the cytosolic and mitochondrial fractions, respectively. The relative ratios of expression in the results of the Western blotting were presented at the bottom of each of the results as relative values of the actin or COX IV or expression. **(C)** The cells were collected and incubated with JC-1 (10 μM) for 20 min at 37°C in the dark. The cells were then washed once with PBS and analyzed by a flow cytometer. The data is expressed as the mean of two independent experiments.

The intrinsic apoptosis pathway is dependent on the release of apoptotic factors such as cytochrome *c* from the mitochondria to the cytosol, which is believed to be an initiator of the caspase cascade. Releasing apoptotic factors into the cytosol requires members of the Bcl-2 family, which is composed of pro- and anti-apoptotic proteins [6,27]. To confirm whether the intrinsic pathway is involved in SCRT-induced apoptosis, the effect of SCRT on the levels of anti-apoptotic Bcl-2, pro-apoptotic protein Bax and cytochrome *c* was monitored. As shown in Figure 5A, the total levels of Bax and Bcl-2 proteins remained unchanged in response to SCRT treatment. However, SCRT treatment decreased cytosolic levels of Bax, while its mitochondrial levels significantly increased after treatment with SCRT in a dose-dependent manner (Figure 5B). Cytochrome *c* was also tested in mitochondrial and cytosolic fractions, as showed in Figure 5B. SCRT treatment caused a marked decrease in mitochondrial cytochrome *c* and a concurrent increase in cytosolic cytochrome *c* (Figure 5B).

The release of cytochrome *c* often involves changes in MMP, and the loss of MMP in cells is one of the early critical events in the intrinsic apoptosis pathway, eventually causing the initiation and activation of apoptotic cascades [28,29]. Therefore, we sought to determine whether SCRT treatment had any effect on the MMP in A549 cells using fluorescent dye JC-1 staining. Figure 5C shows that treatment of A549 cells with SCRT resulted in a dose-dependent increase in the proportion of green fluorescence-positive cells (indicating a loss of mitochondrial membrane potential), confirming that SCRT induced the loss of MMP. These results indicated that SCRT could induce the mitochondrial translocation of Bax and loss of MMP resulting in mitochondrial dysfunction, release of cytochrome *c* to the cytosol, and ultimately, apoptosis induction.

### Effects of SCRT on the expression of cell death receptor (DR)-related proteins and Bid by SCRT in A549 cells

**Figure 6:**
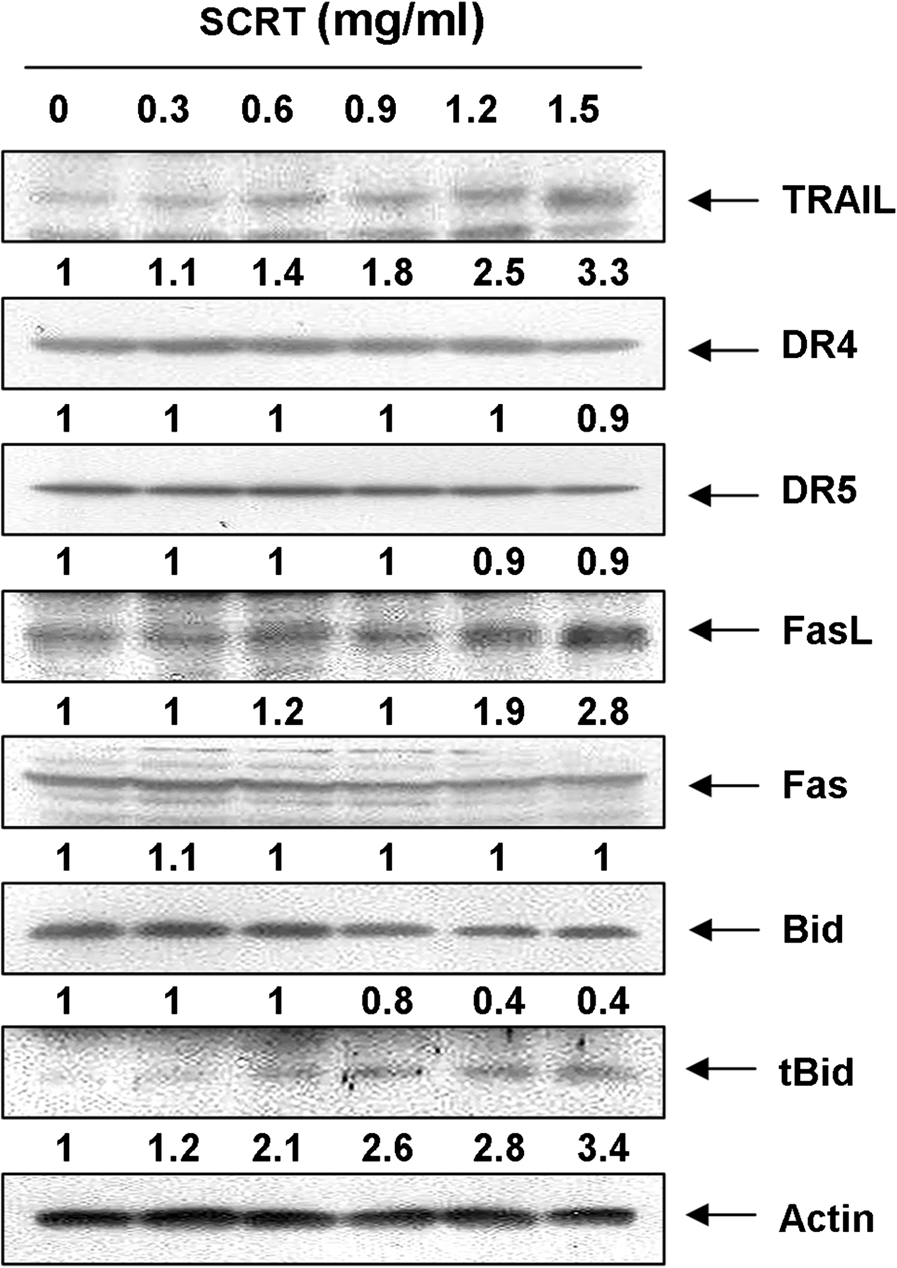
**Effects of SCRT on the expression of DR-related proteins and Bid in A549 cells.** Cells were treated with the indicated concentrations of SCRT for 72 h. The cells were lysed, and the cellular proteins were visualized using the indicated antibodies and an ECL detection system. Actin was used as an internal control. The experiment was repeated three times and similar results were obtained. The relative ratios of expression in the results of the Western blotting were presented at the bottom of each of the results as relative values of the actin expression.

The extrinsic apoptosis pathway begins outside the cell by the binding of death-inducing ligands to their suitable DRs on the cell surface. This is followed by receptor clustering and transmission of the intracellular signals that ultimately result in the destruction of the cell through the activation of caspase-8. Caspase-8 can then activate caspase-3 and cleave Bid, a specific substrate for caspase-8, to form truncated Bid (tBid). Once Bid is cleaved by caspase-8, the tBid translocates to the mitochondrial membrane and enhances cytochrome *c* release, amplifying the mitochondrial-mediated intrinsic pathway [30,31]. Thus, we further investigated whether SCRT affects the extrinsic pathway by analyzing the effects of SCRT on the DR-related proteins and Bid expression. As demonstrated in Figure 6, after SCRT treatment, the levels of DR4, DR5 and Fas were not altered; however, the expression of tumour necrosis factor (TNF)-related apoptosis inducing ligand (TRAIL) and as well as Fas ligand (FasL) strongly increased in a dose-dependent manner. Subsequent Western blot analysis revealed that progressive down-regulation of total Bid protein and accumulation of tBid (Figure 6), presumably resulting from a truncation by caspase-8, occurred in A549 cells treated with SCRT. The results indicate that cytotoxic effects induced by SCRT could be mediated through the DR-mediated apoptosis, thereby accentuating the crosstalk between the intrinsic and extrinsic apoptosis pathways.

### Effects of SCRT on the PI3K/Akt pathway in A549 cells

**Figure 7:**
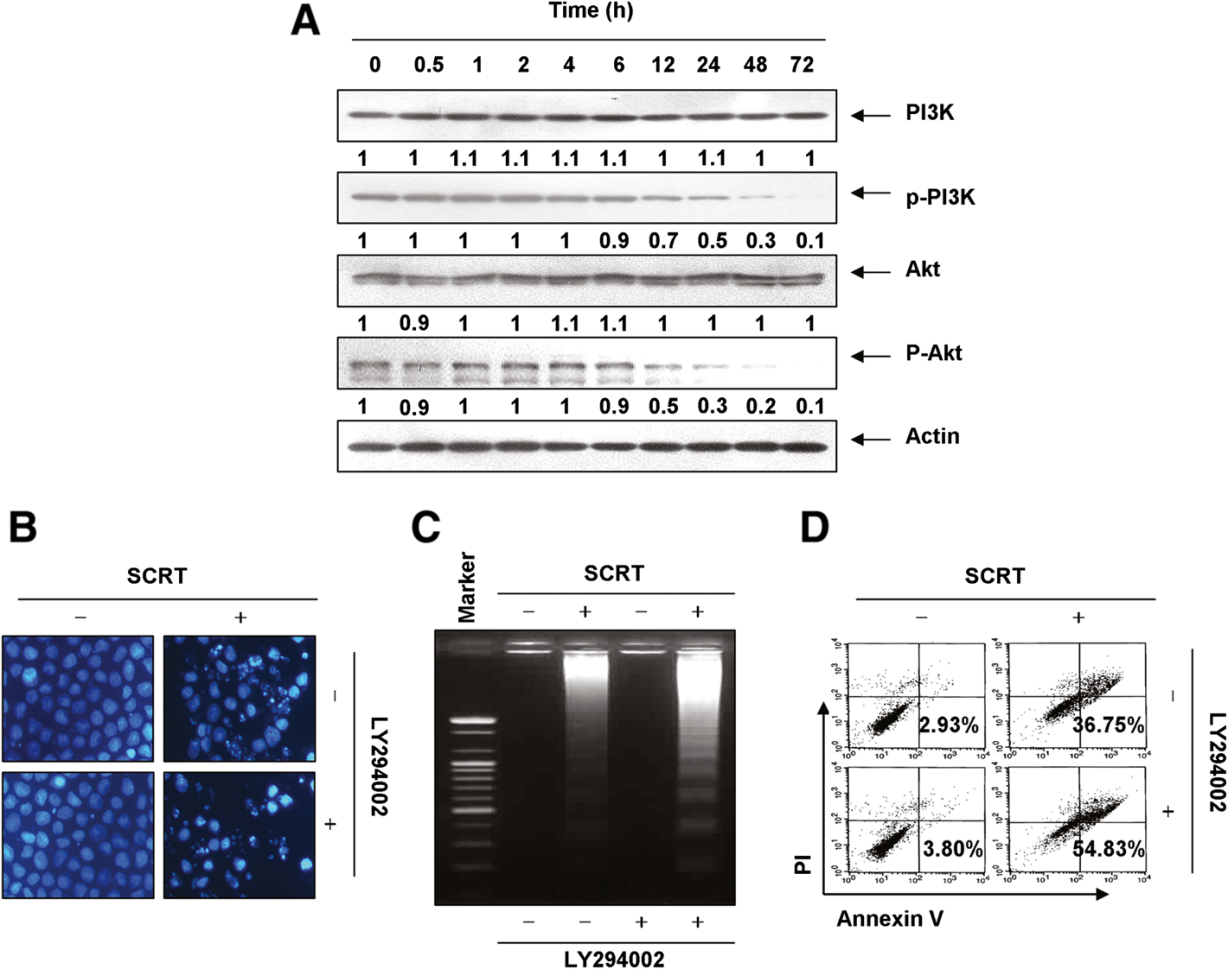
**SCRT triggers apoptosis through an inactivation of PI3K/Akt signaling in A549 cells. (A)** The cells were treated with SCRT (1.5 mg/ml) for the indicated times. Equal amounts of cell lysate were resolved by SDS-polyacrylamide gels, transferred to nitrocellulose membranes, and probed with the anti-p-PI3K, anti-p-Akt, anti-PI3K and anti-Akt antibodies. The proteins were visualized using an ECL detection system. Actin was used as an internal control. The relative ratios of expression in the results of the Western blotting were presented at the bottom of each of the results as relative values of the actin expression. **(B-D)** The cells were pretreated with PI3K inhibitor (LY294002, 50 μM) for 1 h and then treated with SCRT (1.5 mg/ml) for 72 h. **(B)** After staining with DAPI solution, the nuclei were observed under a fluorescent microscope (original magnification 400x). **(C)** The genomic DNA from cells was extracted, separated by agarose gel electrophoresis, and visualized under UV light after staining with EtBr. **(D)** The percentages of apoptotic cells (annexin V^+^ cells) were analyzed using flow cytometric analysis. The data is the mean of the two different experiments.

The PI3K/Akt pathway is one of the most important signaling networks in cell growth and the survival of many cancers [32,33]. To determine the role of SCRT in the PI3K/Akt pathway, we investigated the expression and phosphorylation levels of PI3K and Akt, a downstream effector of PI3K, in A549 cells after treatment with SCRT. As shown in Figure 7A, the phosphorylation levels of PI3K and Akt significantly decreased after SCRT treatment in a time-dependent manner. However, expression levels of total PI3K and Akt proteins remained the same throughout the experiment. To address the possible role of cell viability as a modulation of PI3K/Akt pathway in SCRT-induced apoptosis in A549 cells, we used the PI3K inhibitor, LY294002, for further studies. As shown in Figure 7B-D, treatment of LY294002 markedly increased SCRT-induced apoptosis as demonstrated by nuclear morphology, DNA fragmentation, and annexin V-FITC staining. These results strongly demonstrate that the inactivation of PI3K/Akt signaling pathway is involved in SCRT-induced A549 cell apoptosis.

## Discussion

In the present study, we investigated the effects of SCRT on NSCLC A549 cells and demonstrated that SCRT inhibits A549 cell growth, an effect correlated with apoptosis induction. In our study, chromatin condensation of the nuclei, DNA fragmentation, and an increase of sub-G1 phase, and annexin V-FITC-stained cells demonstrated the apoptosis induction (Figures 1 and 2). We then examined various aspects of the mechanisms of apoptosis induction by SCRT in A549 cells and found SCRT-activated caspase-9 -8, and −3, which are associated with the loss of MMP and degradation of PARP and PLC-γ1. SCRT also down-regulated IAP family proteins, which reportedly block apoptosis directly by binding to and inhibiting several caspases (Figure 3). However, blocking caspase activity by pretreating the cells with a pan-caspase inhibitor significantly prevented SCRT-induced apoptosis and growth inhibition (Figure 4). Therefore, the data support a finding that SCRT-induced apoptosis in A549 cells is caspase-dependent, and both the intrinsic and the extrinsic pathways are activated by SCRT.

Apoptosis, as a programmed cell death, is a rigorous, active, and orderly process of cell death, regulated by numerous gene products to maintain the stability of the intracellular environment. Among them, cytochrome *c* is a small heme protein found loosely associated with the inner membrane of the mitochondrion. The release of cytochrome *c* from the mitochondria into the cytoplasm through an opening of the mitochondrial permeability transition pore (MPTP) followed by a swelling-induced rupture of the mitochondrial outer membrane is an important component in the intrinsic pathway. The protein binds apoptotic protease activating factor-1 (Apaf-1) and activates caspase-9 in the cytoplasm. Once initiated, caspase-9 can go on to activate effector caspases such as caspase-3 and −7, which then cleave many important cellular substrates and causes cell death [5,34,35].

The Bcl-2 protein family is a major regulator of apoptosis, as the balance of pro-apoptotic and anti-apoptotic Bcl-2 family proteins decide the fate of the cell. The proteins are also important regulators of cytochrome *c* release from the mitochondria through the regulation of MPT. For example, the pro-apoptotic protein Bax facilitates the openness of the MPTP and the subsequent cytochrome *c* release, which in turn activates the caspase cascade reaction, eventually resulting in apoptosis. Conversely, the anti-apoptotic protein Bcl-2 protects mitochondria against the loss of function during apoptosis by inhibiting the MPTP’s opening, the Bcl-2-Apaf1-caspase-9 complex, and apotosome, resulting in an anti-apoptosis effect [34,35]. Interestingly, the present data revealed that SCRT did not affect the total expression levels of Bax as well as Bcl-2; however, SCRT treatment resulted in a dose-dependent increase in mitochondrial levels of Bax with a concomitant decrease in the cytoplasmic Bax levels. Our data also clearly showed that SCRT treatment resulted in a dose-dependent increase in the release of cytochrome *c* (Figure 5). Because the release of cytochrome *c* requires mitochondrial membrane insertion and the oligomerization of Bax [22,28,29], the translocation of pro-apoptotic Bax proteins from the cytosol to mitochondria represents a key event for the activation of the intrinsic pathway. Therefore, our data indicated that SCRT induces Bax translocation to mitochondria from the cytosol, leading to the release of cytochrome *c*, apoptosome formation, and finally, induction of apoptosis in A549 cells.

Our results also demonstrate that SCRT activates the death receptor-mediated extrinsic pathway. The extrinsic pathway can be triggered to undergo oligomerization when proper ligands bind their respective death receptors, ultimately recruiting adaptor molecules and caspase-8 into a complex resulting in activated caspase-8. In addition, caspase-8 connects to the intrinsic pathway via cleavage of the Bcl-2 family member Bid to form tBid. Under normal conditions, Bid is located in the cytoplasm in an inactive state; however, tBid can translocate the mitochondrial membrane where it interacts with Bax proteins. This interaction promotes the fusion of Bax and the mitochondria, which results in changes to the configuration of Bax proteins. These changes enhance mitochondrial dysfunction, resulting in the formation of membrane pores and permitting the release of cytochrome *c* to the cytosol, followed by the activation of caspase-9 [28,30]. This event leads to activation of caspase-3 for the induction of apoptosis via a release of cytochrome c to the cytosol [4,8]. Moreover, tBid directly binds to Bcl-2 and thereby inhibits its anti-apoptosis effect [30,31]. Although the levels of death receptors such as DR4, DR5, and Fas remained unchanged in SCRT-treated A549 cells, SCRT notably increased the levels of their ligands including TRAIL and FasL, while a decline of intact Bid occurred concurrently with a pronounced increase of tBid (Figure 6).

On the other hand, PI3K plays a critical role in cell survival and proliferation in collaboration with its major downstream effector Akt. Notably, the aberrant activation of the PI3K/Akt pathway is implicated in the initiation and progression of several types of human malignancies, including NSCLC [36]. Furthermore, deregulation of the PI3K/Akt pathway is associated with resistance to chemotherapeutic agents [32,37]. Meanwhile, anti-apoptotic proteins such as survivin and Bcl-2, and caspase-3 are the downstream target of Akt, and are related to proliferation and apoptosis [38,39]. Many previous studies also have indicated that the pro-apoptotic potential of some anti-cancer agents is highly correlated with the inactivation of the PI3K/Akt pathway [33,40-43]. These observations indicate that the inhibition of the PI3K/Akt signaling cascade can serve as an effective strategy for the treatment of cancers. In the present study, we further studied the possible role of the PI3K/Akt pathway in the SCRT-induced apoptosis of A549 cells and found the levels of phosphorylated PI3K and Akt, were markedly decreased after SCRT treatment (Figure 7A). The total levels of PI3K and Akt were unchanged with SCRT treatment. In addition, there was a significant increase in apoptotic activity when A549 cells were treated with a combination of SCRT and LY294002, a PI3K inhibitor, whereas treatment with LY294002 alone did not affect the growth of A549 cells (Figure 7B-D). This result provides another piece of evidence that SCRT can induce apoptosis by reducing PI3K/Akt pathway activation in A549 cells.

## Conclusions

In conclusion, we described how SCRT significantly inhibits the growth of NSCLC A549 cells and induces cell apoptosis *in vitro*. SCRT treatment increases the loss of MMP and translocalization of Bax to the mitochondria, and leads to cytochrome *c* release to the cytosol and activation of caspase-9 and −3, resulting in the activation of the intrinsic apoptosis pathway. SCRT also activates the extrinsic apoptosis pathway by increasing both TRAIL and FasL, resulting in the activation of caspase-8 and Bid truncation. Thereby the mechanism of action of SCRT is related to the activation of the extrinsic as well as the intrinsic apoptosis pathways, amplifying mitochondrial signaling. In addition, co-treatment of LY294002, a PI3K inhibitor, significantly increased SCRT-induced-apoptosis indicating that the PI3K/AKT pathway might have a survival role in SCRT-induced A549 cell apoptosis. To explore this rule, the pathway must be blocked while the cells are treated with SCRT. Although *in vivo* anti-cancer activity and deep molecular mechanisms need further investigation, based on the results of this study, we believe SCRT should continue to be developed as a possible new therapeutic treatment for NSCLC.
